# Multi-Pitch Liquid Crystal Filters with Single Layer Polymer Template

**DOI:** 10.3390/polym13152521

**Published:** 2021-07-30

**Authors:** Zhikang Zhu, Yao Gao, Jiangang Lu

**Affiliations:** National Engineering Lab for TFT-LCD Materials and Technologies, Department of Electronic Engineering, Shanghai Jiao Tong University, Shanghai 200240, China; ZhiKang_Zhu@sjtu.edu.cn (Z.Z.); gaoyao123@sjtu.edu.cn (Y.G.)

**Keywords:** polymer template, single layer, liquid crystal filter

## Abstract

Multi-reflective peak and bandwidth scalable liquid crystal (LC) filters were investigated. By refilling a cholesteric LC (CLC) whose chiral pitch is different to the target template into a blue phase LC (BPLC) template, a multi-reflective peak single layer LC filter can be fabricated. With multiple templating and refilling processes, the number of reflective peaks can be further increased. Moreover, by refilling the CLCs of designed chiral pitch into a CLC template sequentially, a bandwidth scalable single layer CLC filter can be fabricated. The LC filters show great potential applications in optical communication, display, and LC lasing.

## 1. Introduction

**Figure 1:**
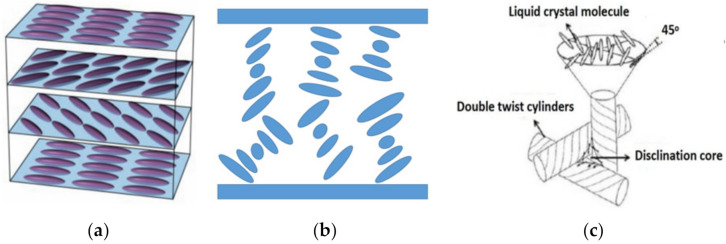
The arrangements of LC molecules in (**a**) planar texture state CLC; (**b**) focal conic CLC; (**c**) BPLC.

Cholesteric liquid crystals (CLCs) have one-dimensional twist structures with a continuous periodic distribution of molecules through the planar orientation of the interface [1]. The arrangements of LC molecules in CLCs consist of planar and focal conic states, which are shown in Figure 1a,b. When the linearly polarized light is incident on the cholesteric liquid crystal, its polarization direction is gradually twisted—that is, the plane of vibration of polarized light is rotated by virtue of the helical structure of CLCs [2,3]. However, when unpolarized light is incident, under the action of the helical structure composed of cholesteric liquid crystal molecules, the phenomenon of left or right rotation will be generated in the electric vibration vector of the light wave, and the light is divided into circularly polarized light with left/right rotation characteristics. The circularly polarized incident light with the same handedness as that of the CLCs is reflected, while that with the opposite handedness is transmitted [4]. The blue phase liquid crystals (BPLCs) appear as a regular array of double twist cylinders (DTCs) and the disclination lines among them exist in a narrow temperature range between the isotropic phase and cholesteric phase [5,6,7,8]. The arrangement of LC molecules in BPLCs is shown in Figure 1c. Due to the helical twist structures, both CLCs and BPLCs show Bragg reflection properties [9,10,11,12,13].

Recently, a templating technology was proposed to improve the thermal stability of CLCs and BPLCs [14,15]. The templating technology is a method that can convert the achiral nematic LC(NLC) to CLC or BPLC with twist structure and improve thermal stability. The helical twist structure was obtained by chiral dopant (such as R5011, S811, and R811) which induces the twist deformation of the molecular director in an achiral LC. Herein, a chiral polymer network was formed by combining the chiral dopant with other materials, and by refilling the NLC into the CLC template and BPLC template, respectively, the template-CLC (T-CLC) and template-BPLC (T-BPLC) with chiral twist structure can be achieved, respectively, which differ by the self-assembly process. Due to the Bragg reflection property and the templating technology, LC filters are a popular research direction, including multi-wavelength and bandwidth tunable LC filters [16,17]. However, most multi-wavelength and bandwidth tunable LC filters are formed based on the integration of multi-layer structures, which have complicated preparation processes and are not conducive to the large-scale application of chiral helical structure liquid crystals [18,19,20]. In this paper, the reconstructing capability of the CLC, BPLC template was illustrated. Moreover, multi-wavelength BPLC filters and bandwidth tunable CLC filters were fabricated based on the above conclusions. A single layer preparation method for twist structure LC filters was proposed. The results clearly showed that the method had good scalability, and theoretically it was possible to obtain LC filters with more central reflection wavelengths and tunable bandwidth, which are expected to be applied to optical communication and display.

## 2. Materials and Methods

### 2.1. Materials Preparation

In order to obtain our desired LC filters, the materials required for the experiments mainly consisted of the following components: a positive nematic LC (BPH006, Jiangsu Hecheng Display Technology Co., Ltd.(HCCH), Nanjing, Jiangsu, China), chiral dopant (R5011, Nanjin Murun, Nanjing, Jiangsu, China), monomer (TMPTA, Shanghai Macklin, Shanghai, China), cross-linker (C3M, HCCH, Nanjing, Jiangsu, China), and photo-initiator (IRG184, HCCH, Nanjing, Jiangsu, China). The chemical structures are shown in Figure S2 (Supplementary Materials). In order to obtain LC filters with two reflection peaks, we need to obtain polymer-stabilized blue phase liquid crystal (PS-BPLC) precursors and CLC; PS-BPLC precursors are used to make the blue phase template, and CLC is used as a refilling material, considering that two reflection peaks of the dual-wavelength LC filter should be separated, and the reflection bandwidth of CLC is wider, so the center wavelength of the transmittance spectrum of CLC should be far from the intrinsic reflection peak of BPLC template. The central wavelength of their transmittance spectra can be adjusted by changing the ratio of chiral dopant in the material system, as shown in Table 1.

To obtain LC filters with three reflection peaks, we need to obtain PS-BPLC precursors, polymer-stabilized cholesteric liquid crystal (PS-CLC) precursors 1 and CLC 2; the PS-BPLC precursors are used to make the intrinsic blue phase template, the PS-CLC 1 precursors are used to fabricate the double-peaked template, and the CLC 2 is used to form the third reflection peak. The ratio of PS-BPLC precursors, PS-CLC precursors 1, and CLC 2 are shown in Table 2.

To obtain bandwidth tunable LC filters, we need to obtain two PS-CLC precursors and one CLC. The PS-CLC 1 precursor is used to make the intrinsic CLC 1 template, the PS-CLC 2 precursor is used to make the template for the primary broadening of the bandwidth, and CLC 3 is used as the refilling material for the secondary broadening of the bandwidth. The ratios of two kinds of PS-CLC precursors and CLC 3 are shown in Table 3. In order to obtain the different central wavelength LC filters, the ratios of precursors or CLC are different, and the PS-CLC 1, PS-CLC 2, CLC 2, and CLC 3 are named according to the different ratios.

### 2.2. Fabricating Process

**Figure 2:**
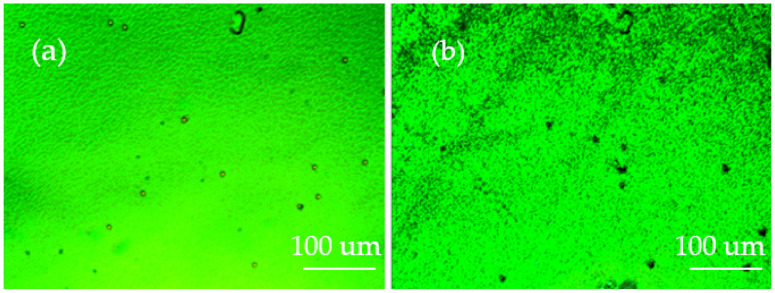
The surface morphology of (**a**) PS-BPLC and (**b**) T-BPLC.

In order to obtain the BPLC template, we first added the material to a container according to the above ratio, and then put the container on the thermostatic magnetic stirrer. Raising the temperature to 80 °C and stirring for about 10 min, the material showed a transparent liquid shape, which means the material has been stirred well and the precursor of PS-BPLC was obtained. Then, we put the precursor on a temperature controller (HCS302, Intec, Boulder, Co., USA) and set the temperature controller to 80 °C in order to ensure that the precursor was added to the LC cell in an isotropic state, and used the capillary phenomenon to infuse the precursor into the anti-parallel LC cell with a thickness of 8 μm and area of 25 mm × 20 mm. Then, we observed the change of the LC phase state by lowering the temperature at a rate of 0.5 °C/min. The temperature range of the LC corresponding to the phase state was determined. Finally, we obtained the PS-BPLC by UV exposure (wavelength: 365 nm, exposure time: 10 min, exposure intensity: 3 mw/cm^2^). The platelet texture of the PS-BPLCs was observed under a polarized optical microscope (POM, XPL-30TF, Shanghai WeiTu Optics & Electron Technology Co., Ltd., Shanghai, China), as shown in Figure 2a. Then, the PS-BPLC cells were immersed in the organic solvent, acetone, for about 48 h to wash out the LC, chiral dopant, unreacted monomers, and photoinitiator. After evaporating on the temperature controller (temperature: 80 °C), the BPLC templates were obtained and the blue phase texture of templated-BPLC (T-BPLC) could be observed, as shown in Figure 2b. During to the fact that the central wavelength of PS-BPLC is in range of green, Figure 2 is green, which is irrelevant to the phase state. If the central wavelength is in range of blue or red, the texture figure will be blue or red.

A CLC whose central wavelength is much shorter than that of the BPLC template was refilled into the template at 80 °C and cooled down to 30 °C at a rate of 0.5 °C/min to make a filter with two central reflection wavelengths. To obtain an LC filter with three central reflection wavelengths, at first, a PS-CLC precursor, whose central wavelength is much shorter than that of the BPLC template, was refilled into the BPLC template at 80 °C. Then, after the UV exposure and the wash out process, a template with both BPLC and CLC structure was fabricated. Finally, the long central wavelength CLC, which is far away from the peak of the BPLC template, was refilled into the dual peak template at 80 °C and cooled down to 30 °C at a rate of 0.5 °C/min, and the tri-peak chiral LC filter was fabricated.

**Figure 3:**
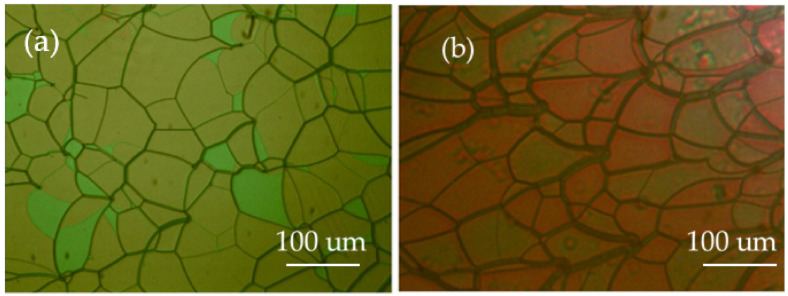
The surface morphology of (**a**) PS-CLC and (**b**) T-CLC.

**Figure 4:**
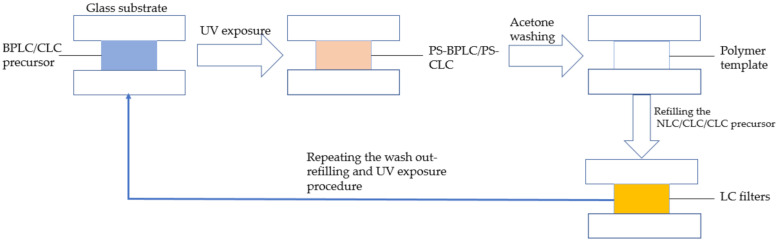
Flow chart of multiple refilling process.

In order to obtain a bandwidth tunable CLC filter, first we made a CLC template by the same template process as the BPLC template. The cholesteric texture of PS-CLC and T-CLC could be observed, as shown in Figure 3a,b. The molecular structures in our experiment are shown in Figure S1 (Supplementary Materials). Then, the short central wavelength PS-CLC precursor with a close peak distance to the intrinsic CLC template was refilled at 80 °C, and the templating process was repeated. Finally, after the long central wavelength CLC with a close peak distance to the intrinsic CLC template was refilled into the template and cooled down to 30 °C at a rate of 0.5 °C/min, the bandwidth tunable CLC filter was fabricated. The fabrication process of the multiple LC refilling is shown in Figure 4.

**Figure 5:**
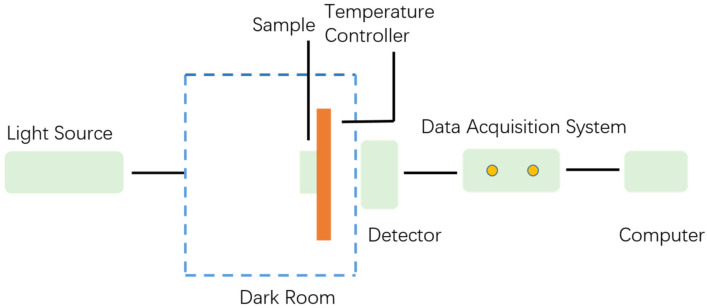
Schematic diagram of transmittance acquisition device.

The spectral characteristics measurement system is shown in Figure 5. A tungsten bromide lamp was used as a non-polarized light source, providing a spectrum in the visible band. The light source signal is incident on the device, and the light intensity of the incident light signal is collected by the detector of the data acquisition system (DCS300PA, Zolix), and the transmittance curve is finally obtained by calculation. While the transmittance intensity of the transmittance spectra may be reduced by scattering or absorbing, the central wavelength, bandwidth, and the maximum transmissivity/reflectivity of the transmittance and reflectance spectra almost had the same values.

## 3. Results and Discussions

### 3.1. Filtering Characteristics of Dual-Wavelength Single Layer LC Filter

**Figure 6:**
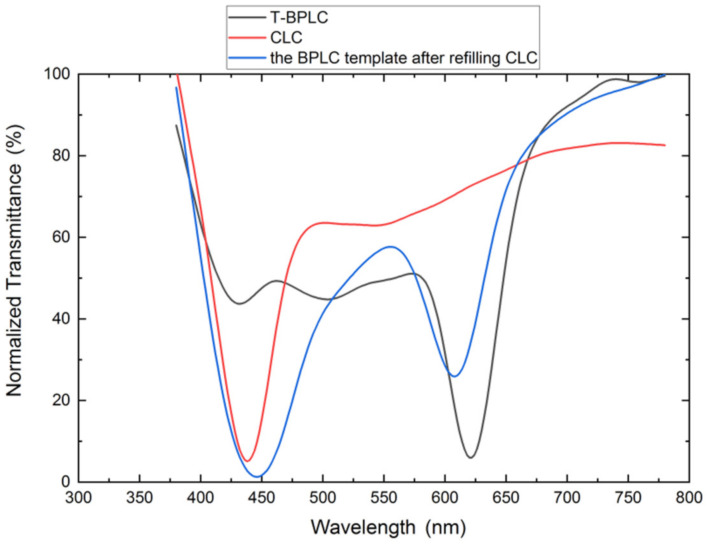
Transmittance spectra of T-BPLC, CLC, and dual-wavelength LC filter.

A dual-wavelength LC filter was obtained by filling a CLC whose central wavelength is far from that of the intrinsic BPLC template into the template, and its transmittance spectrum is shown in Figure 6. The central wavelength of the templated BPLC and the CLC are 620 nm (black line) and 438 nm (red line), and the FWHMs are 54 nm and 62 nm, respectively; the two reflective peaks of the dual-wavelength LC filter are 608 nm and 446 nm (blue line). We may see that the two reflective peaks of the dual-wavelength LC filter are close to the central wavelength of templated BPLC and CLC, and only the band width increases a little. The results show that the dual-wavelength LC filter fabricated by the multi-refilling process maintains a good consistency with the design parameters.

### 3.2. Filtering Characteristics of Three-Wavelength Single Layer LC Filter

**Figure 7:**
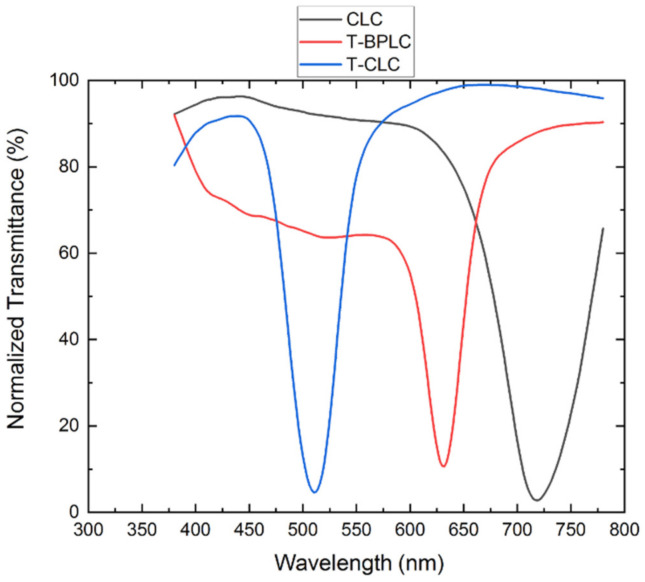
Transmittance spectra of T-CLC, T-BPLC and CLC2.

The transmittance spectra of T-BPLC, T-CLC, and CLC were measured, and the results are shown in Figure 7. The central wavelength of the templated BPLC is 630 nm (red line) and its FWHM is 46 nm. The central wavelength of the templated CLC is 510 nm (blue line) and its FWHM is 54 nm. The central wavelength of the CLC is 720 nm (black line) and its FWHM is 80 nm. The three kinds of LC materials are designed to fabricate the three reflection peaks of the LC filter, as their central wavelengths are far from each other and their FWHMs are relatively narrow.

**Figure 8:**
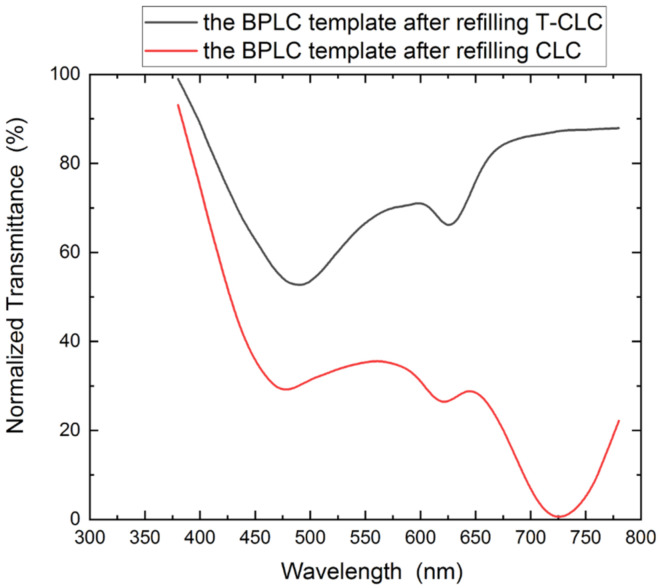
Transmittance spectra of the blue phase template after the first refill and the second refill.

Then, a PS-CLC precursor was refilled into the BPLC template, and a dual-wavelength LC filter was successfully fabricated after exposure to ultraviolet light. The transmittance spectrum is shown as the black line in Figure 8. Different from the two-wavelength LC filter we fabricated above, the blue phase template was filled with PS-CLC, and the LC template with two reflection peaks was fabricated by acetone soaking. Finally, we refilled CLC into the dual-wavelength template to obtain a three-wavelength LC filter, and its transmittance spectrum is shown as the red line in Figure 8. The filter has a total of three central wavelengths, namely 620 nm, 481 nm, and 725 nm. The central wavelength of 620 nm is close to the central wavelength of the intrinsic blue phase template, the central wavelength of 481 nm is close to the peak of the T-CLC, and the central wavelength of 725 nm is close to the central wavelength of the CLC. However, the FWHM of the waveform has been broadened to a certain extent, and the waveform has a relatively obvious downward shift. The reason for this phenomenon may be due to the wide bandwidth of the cholesteric LC and the large reflection peak of the long wavelength. The central wavelength remains basically unchanged, which also indicates that the three-wavelength LC filter fabricated by the above method has good stability and the method has good scalability. The position of the central wavelength can be designed according to the requirements.

### 3.3. Filtering Characteristics of Single Layer CLC Filter with Tunable Bandwidth

**Figure 9:**
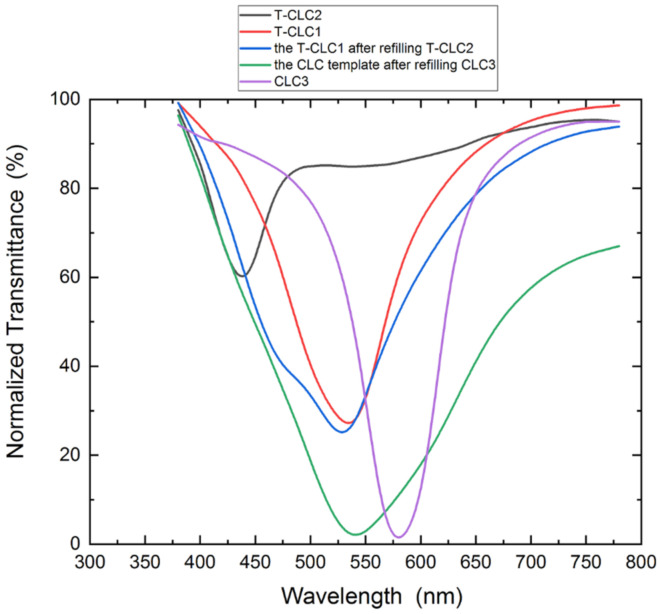
Bandwidth tunable LC filter reflection spectrum.

Firstly, the transmittance spectra of the T-CLC1, the T-CLC2, and the CLC were measured, as shown in Figure 9. Among them, the central wavelength of the T-CLC1 is 534 nm (red line), its FWHM is 111 nm, and the central wavelength of the T-CLC2 is 438 nm (black line), and its FWHM is 61 nm, the central wavelength of the CLC3 is 579 nm (purple line), and its FWHM is 82 nm. The central wavelengths of the T-CLC1, T-CLC2, and CLC3 are close to each other, which is suitable for the production of tunable bandwidth LC filters.

Then, we refilled the PS-CLC2 precursor into the CLC template 1, and successfully prepared an LC filter with a wide bandwidth after exposure to ultraviolet light. The transmittance spectrum is shown as the blue line in Figure 9, its FWHM is 162 nm. The FWHM of theT-CLC1 and the T-CLC2 are 111 nm and 61 nm, respectively, and their sum is 172 nm, which is close to the FWHM of the above device. Finally, an LC template after the first refill was fabricated by acetone soaking, and CLC3 was refilled into the template to prepare a broadband LC filter. The transmittance spectrum is shown in the green line of Figure 9, and its FWHM is 218 nm. The FWHM of the CLC filter after the first refilling and of the CLC3 are 162 nm and 82 nm, respectively, and their sum is 244 nm, which is close to the FWHM of the cholesteric LC filter after the second refill. The results show that the LC filter with tunable bandwidth can be fabricated well through the process of multiple LC refills. Moreover, it can be seen from the waveform observation that the center wavelength of the bandwidth tunable LC filter fabricated by the multiple LC refill process is consistent with the intrinsic peak center wavelength of the template, which verifies the stability of the method. When using the above method to fabricate a bandwidth tunable LC filter, the central wavelength of the intrinsic peak of the template should be calculated first, because this determines the central wavelength of the final device. Additionally, the fabrication method is scalable, which means the bandwidth of CLC can be broadened many times, and the bandwidth of the filter depends on the refilling times and the refilled LC materials.

## 4. Conclusions

A multi-wavelength chiral structure LC filter and a bandwidth tunable CLC filter with a single layer structure was fabricated by the multiple wash out–refilling process. The experimental results show that the maximum deviation between the center wavelength of the multi-wavelength LC filter and the designed center wavelength is only 16 nm, and the FWHM of the tunable CLC filter can be broadened by 96% compared with the original FWHM of the CLC filter, 111 nm, and can be broadened continuously. Theoretically, the bandwidth of CLCs can be expanded many times to prepare a CLC filter with large bandwidth covering the whole band from ultraviolet to infrared. Compared with the multi-wavelength BPLC filter of the multi-layer structure, the single layer structure multi-wavelength chiral structured LC filter and the bandwidth tunable CLC filter can significantly simplify the fabrication process and the device structure. Moreover, the templating technology with multiple wash out–refilling and UV exposure processes shows good stability and extensibility, and offers potential applications in display and optical communication. It is worth noting that only the transmission spectra of LC filters, and how multi-wavelength LC filters and bandwidth tunable CLC filters are fabricated, was reported, while the applications of LC filters under electric fields constitute our following studies.

## Figures and Tables

**Table 1:** Composition ratio of dual-wavelength LC filter (wt %).

	BPH006	R5011	TMPTA	C3M	IRG184
PS-BPLC	84.5	3.4	5.12	6.88	0.1
CLC	97.5	2.5			

**Table 2:** Composition ratio of three-wavelength LC filter (wt %).

	BPH006	R5011	TMPTA	C3M	IRG184
PS-BPLC	84.5	3.4	5.12	6.88	0.1
PS-CLC 1	85.9	2.0	5.12	6.88	0.1
CLC 2	98.5	1.5			

**Table 3:** Composition ratio of bandwidth tunable LC filter (wt %).

	BPH006	R5011	TMPTA	C3M	IRG184
PS-CLC 1	86.1	1.8	5.12	6.88	0.1
PS-CLC 2	85.7	2.2	5.12	6.88	0.1
CLC 3	98.3	1.7			

## Data Availability

The data that support the findings of this study are available from the corresponding author upon reasonable request.

## Supplementary Materials

The following are available online at https://www.mdpi.com/article/10.3390/polym13152521/s1, Figure S1: The molecular structures of templates and procedure for preparing templates, Figure S2: The chemical structure of materials.
