# Supplementary material for: Multi-Pitch Liquid Crystal Filters with Single Layer Polymer Template
Source: Polymers (Basel). 2021 Jul 30;13(15):2521. doi: 10.3390/polym13152521 (PMC8348528; doi:10.3390/polym13152521)
Supplement: Supplementary file 1 [file polymers-13-02521-s001.zip › polymers-1283369-supplementary.pdf]

polymers

MDPI

Communication

Multi-Pitch Liquid Crystal Filters with Single Layer Polymer Template

Zhikang Zhu, Yao Gao and Jiangang Lu *

National Engineering Lab for TFT-LCD Materials and Technologies, Department of Electronic Engineering, Shanghai Jiao Tong University, Shanghai 200240, China; ZhiKang_Zhu@sjtu.edu.cn (Z.Z.); gaoyao123@sjtu.edu.cn (Y.G.)
* Correspondence: lujg@sjtu.edu.cn

Abstract: Multi-reflective peak and bandwidth scalable liquid crystal (LC) filters were investigated. By refilling a cholesteric LC (CLC) whose chiral pitch is different to the target template into a blue phase LC (BPLC) template, a multi-reflective peak single layer LC filter can be fabricated. With multiple templating and refilling processes, the number of reflective peaks can be further increased. Moreover, by refilling the CLCs of designed chiral pitch into a CLC template sequentially, a bandwidth scalable single layer CLC filter can be fabricated. The LC filters show great potential applications in optical communication, display, and LC lasing.

Keywords: polymer template; single layer; liquid crystal filter

Citation: Zhu, Z.; Gao, Y.; Lu, J. Multi-Pitch Liquid Crystal Filters with Single Layer Polymer Template. *Polymers* **2021**, *13*, 2521. https://doi.org/10.3390/polym13152521

Academic Editor: Rafael Muñoz-Espí

Received: 17 June 2021
Accepted: 28 July 2021
Published: 30 July 2021

Publisher's Note: MDPI stays neutral with regard to jurisdictional claims in published maps and institutional affiliations.

Copyright: © 2021 by the authors. Licensee MDPI, Basel, Switzerland. This article is an open access article distributed under the terms and conditions of the Creative Commons Attribution (CC BY) license (http://creativecommons.org/licenses/by/4.0/).

Supplementary Materials

Figure S1 shows the molecular structures of LC cell and a procedure for preparing the templates. Figure S1(a) shows molecular structure and Figure S1(b) shows the chiral polymer network chain in cell after UV irradiation. Figure S1(c) shows the polymer network after wash-out process.

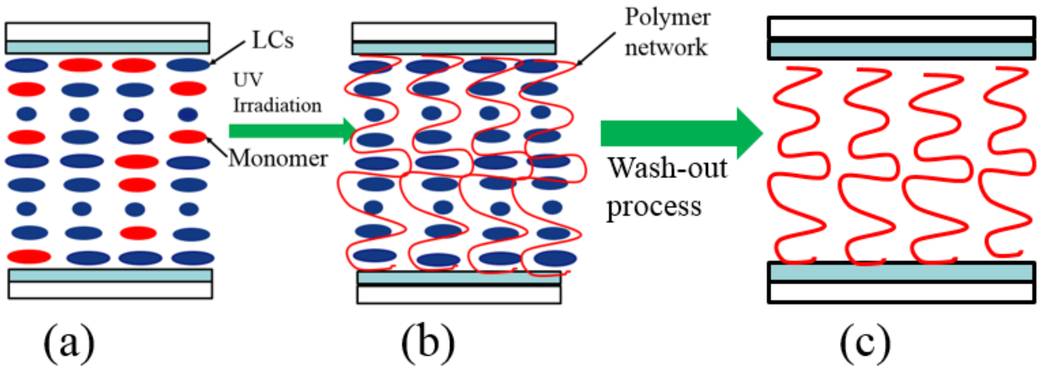

Figure S1. The molecular structures of templates and procedure for preparing templates.

The chemical structure of materials used in our experiment are shown in Figure S2.

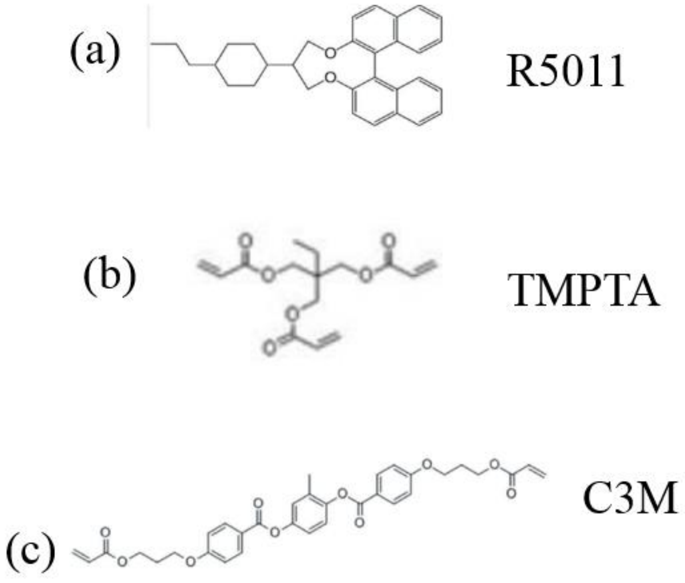

Figure S2. The chemical structure of materials.
